# Protein palmitoylation is critical for the polar growth of root hairs in Arabidopsis

**DOI:** 10.1186/s12870-015-0441-5

**Published:** 2015-02-13

**Authors:** Yu-Ling Zhang, En Li, Qiang-Nan Feng, Xin-Ying Zhao, Fu-Rong Ge, Yan Zhang, Sha Li

**Affiliations:** State Key Laboratory of Crop Biology, College of Life Sciences, Shandong Agricultural University, Tai’an, 271018 China

**Keywords:** Polar growth, 2-bromopalmitate, TIP1, Actin microfilaments, Endocytosis

## Abstract

**Background:**

Protein palmitoylation, which is critical for membrane association and subcellular targeting of many signaling proteins, is catalyzed mainly by protein S-acyl transferases (PATs). Only a few plant proteins have been experimentally verified to be subject to palmitoylation, such as ROP GTPases, calcineurin B like proteins (CBLs), and subunits of heterotrimeric G proteins. However, emerging evidence from palmitoyl proteomics hinted that protein palmitoylation as a post-translational modification might be widespread. Nonetheless, due to the large number of genes encoding PATs and the lack of consensus motifs for palmitoylation, progress on the roles of protein palmitoylation in plants has been slow.

**Results:**

We combined pharmacological and genetic approaches to examine the role of protein palmitoylation in root hair growth. Multiple PATs from different endomembrane compartments may participate in root hair growth, among which the Golgi-localized PAT24/TIP GROWTH DEFECTIVE1 (TIP1) plays a major role while the tonoplast-localized PAT10 plays a secondary role in root hair growth. A specific inhibitor for protein palmitoylation, 2-bromopalmitate (2-BP), compromised root hair elongation and polarity. Using various probes specific for cellular processes, we demonstrated that 2-BP impaired the dynamic polymerization of actin microfilaments (MF), the asymmetric plasma membrane (PM) localization of phosphatidylinositol (4,5)-bisphosphate (PIP_2_), the dynamic distribution of RabA4b-positive post-Golgi secretion, and endocytic trafficking in root hairs.

**Conclusions:**

By combining pharmacological and genetic approaches and using root hairs as a model, we show that protein palmitoylation, regulated by protein S-acyl transferases at different endomembrane compartments such as the Golgi and the vacuole, is critical for the polar growth of root hairs in Arabidopsis. Inhibition of protein palmitoylation by 2-BP disturbed key intracellular activities in root hairs. Although some of these effects are likely indirect, the cytological data reported here will contribute to a deep understanding of protein palmitoylation during tip growth in plants.

**Electronic supplementary material:**

The online version of this article (doi:10.1186/s12870-015-0441-5) contains supplementary material, which is available to authorized users.

## Background

Protein palmitoylation, or S-acylation, is a reversible post-translational modification that adds a 16-carbon saturated palmitate group to the sulfhydryl group of a cysteine to form a thioester [1-3]. Such modifications affect protein trafficking, protein interactomes and protein stability [1-3]. Palmitoylation, usually combined with other lipid modifications such as N-myristolyation and prenylation, provides a hydrophobic membrane anchor on otherwise soluble proteins, enhancing their membrane association [1,2,4]. Transmembrane (TM) proteins, such as receptor kinases and transporters, can also be modified by palmitoylation, which often affects their subcellular targeting and dynamic sorting among different endomembrane compartments [1].

Palmitoyl proteomics indicated that eukaryotes contain a large number of palmitoylated proteins [1,5-7]. Most of palmitoylated proteins, such as small GTPases, receptor tyrosine kinases, transporters, and *N*-ethylmaleimide-sensitive factor-activating protein receptors (SNAREs), are involved in cell signaling and intracellular transport [1,5-7]. In plants, a few proteins have been experimentally demonstrated to be modified by palmitoylation, including ROP GTPases [8-10], CBLs [11,12], subunits of heterotrimeric G proteins [13,14], protein phosphatases [15], and the receptor kinase FLAGELLIN-SENSING 2 [5]. Modification of these key signaling proteins implies that palmitoylation plays crucial roles in plant growth.

Three types of enzymes are reported to catalyze protein palmitoylation [3], among which protein S-acyl transferases (PATs), characterized by an evolutionarily conserved and catalytically critical Asp-His-His-Cys (DHHC) motif within a cysteine-rich domain, play dominant roles [1,3]. DHHC-type PATs are encoded in all eukaryotic genomes [1]. As transmembrane™ proteins, PATs are found at different endomembrane compartments including the Golgi, endoplasmic reticulum (ER), the plasma membrane (PM), and vacuolar membrane in yeast [16]. Recently, it was shown that Arabidopsis PATs have more diverse targeting than their yeast or metazoan counterparts, at the PM, the Golgi, ER, the tonoplast, or various vesicles of distinct identities [17]. Two plant *PAT*s have been functionally characterized [12,18]. Arabidopsis *TIP GROWTH DEFECTIVE1* (*TIP1*)/*PAT24* encodes a PAT with ankyrin repeats, whose mutations result in defective growth both in tip-growing cells, i.e. root hairs and pollen tubes, and in non-tip-growing cells [18-20]. We recently characterized a tonoplast-localized PAT, PROTEIN S-ACYL TRANSFERASE10 (PAT10), which is critical for vacuolar function [12]. In the *pat10* mutants, several CBLs lost their palmitoylation-dependent tonoplast association [12], suggesting that these CBLs are the substrates of PAT10.

Despite the importance of protein palmitoylation for plant growth, progress in understanding plant PAT functions has been slow due to redundancy and overlapping substrate specificity [1,2]. We report here that protein palmitoylation regulates the polar growth of root hairs by using a pharmacological approach in combination with genetics. Root hair growth requires the dynamic distribution of intracellular activities such as actin MF [21-24] and membrane trafficking [24-30]. Many proteins mediating such dynamic activities are likely regulated by palmitoylation based on evolutionary conservation [1] or results from palmitoyl proteomics [5]. Thus, root hairs represent an excellent single cell system to study the effect of protein palmitoylation on multiple intracellular activities.

We show that the Golgi-localized TIP1 plays a major role while the tonoplast-localized PAT10 plays a minor role in the polar growth of root hairs. By application of 2-bromopalmitate (2-BP) that specifically inhibits protein palmitoylation *in vitro* [31] and *in planta* [11,12,18], we show here that inhibiting palmitoylation directly or indirectly impaired actin MF polymerization, abolished the restricted PM localization of PIP_2_, disrupted the dynamic distribution of RabA4b-positive post-Golgi secretion, and inhibited vacuolar trafficking, resulting in defective root hair growth. Thus our results demonstrate the role of protein palmitoylation in intracellular activities that contribute to cell morphogenesis in root hairs and provide experimental evidences to narrow down potential PAT targets in plants.

## Results

### Optimization of 2-BP treatment on root hair growth

**Figure 1:**
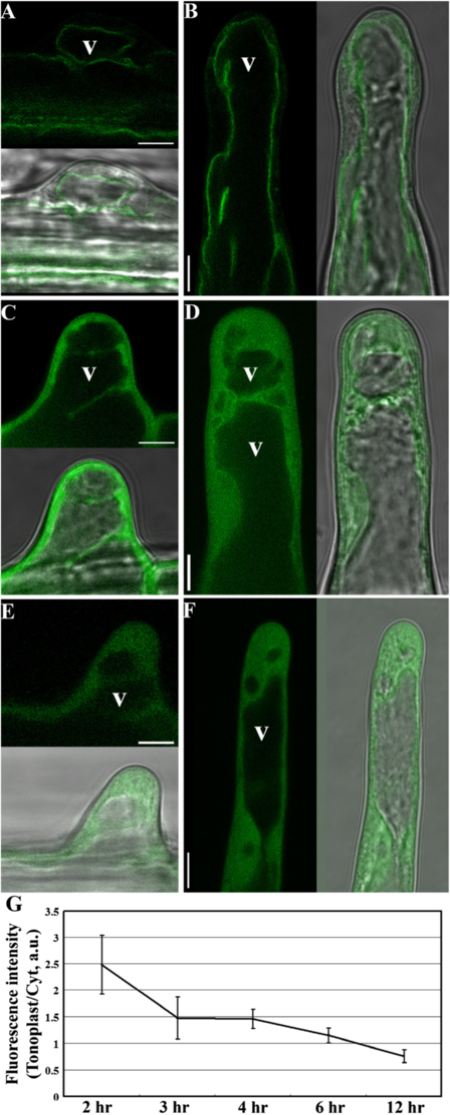
**2-BP abolished the tonoplast localization of CBL2 in root hairs. A**-**D**. 4 DAG seedlings of *Pro*_*UBQ10*_:CBL2-RFP transgenic plants treated with either DMSO (A, B) or 2-BP (C, D) for 4–12 hr before visualization. **E**-**F**. 4 DAG seedlings of *Pro*_*UBQ10*_:CBL2-RFP;*pat10-2* transgenic plants treated with DMSO for 4–12 hr before imaging. Representative initiating (A, C, E) or elongating (B, D, F) root hairs are shown. V indicates vacuole. **G**. Quantification of CBL2-RFP distribution in the tonoplast v.s. the cytoplasm (Tonoplast/Cyt) at different time points after 2-BP treatment. a.u. stands for arbitrary fluorescence units. Bars = 7.5 μm.

To examine cellular processes affected by 2-BP in root hairs, it was necessary to develop a suitable treatment regime that would reveal the effects of inhibiting protein palmitoylation on cellular processes without causing severe cellular damages. To do so, we utilized the subcellular localization of CBL2 as an indicator for the effective inhibition of palmitoylation. CBL2 dissociated from the tonoplast and moved to the cytoplasm when its key palmitoylation site was mutated [11] or in the *pat10* mutants [12]. Based on previous studies [11,12,18], we added 2-BP at a final concentration of 10 μM to 50 μM to *Pro*_*UBQ10*_:CBL2-RFP transgenic seedlings 4 days after germination (DAG) in a hypotonic MS solution, to determine the effects on the tonoplast association of CBL2. Because 2-BP was dissolved in dimethyl sulfoxide (DMSO), equivalent volumes of DMSO were applied as controls in which no phenotypic consequences were detected over the time course of the experiments (Figure 1A,B). As an additional control, we also introduced the same *Pro*_*UBQ10*_:CBL2-RFP transgene into *pat10-2* by crosses [12], in which CBL2 was rendered cytosolic (Figure 1E,F). As expected, 2-BP treatment resulted in gradual relocalization of CBL2 from the tonoplast to the cytoplasm of root hairs (Figure 1). Root hairs incubated with 10 μM 2-BP for 2–3 hrs showed the most substantial reduction of CBL2 at the tonoplast (Figure 1G) and at 12 hrs showed complete absence of tonoplast-CBL2 (Figure 1). Increasing 2-BP concentration from 10 μM to 50 μM did not substantially accelerate the tonoplast dissociation of CBL2 but induced extensive vacuolation (Additional file 1: Figure S1). Based on these results, we used 10 μM 2-BP on root hairs and examined cellular processes from 2 to 12 hrs after 2-BP application for further experiments.

### Root hair growth was impaired by 2-BP

**Figure 2:**
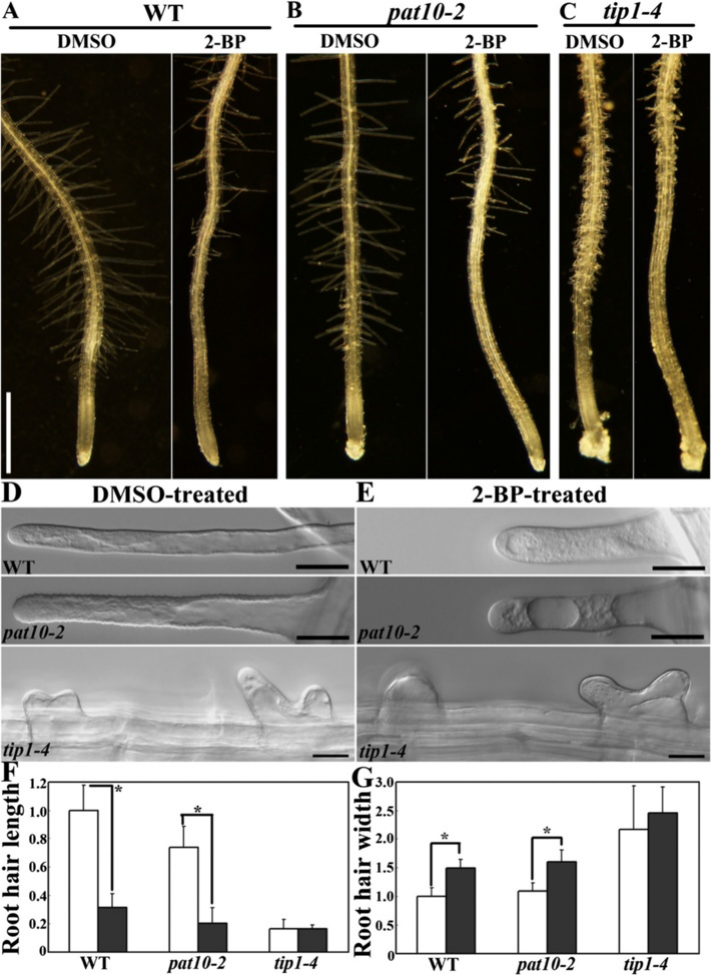
**2-BP impaired root hair growth. A**. Primary roots from 4 DAG seedlings of wild type treated with either DMSO or 2-BP. **B**. Primary roots from 4 DAG seedlings of *pat10-2* treated with either DMSO or 2-BP. **C**. Primary roots from 4 DAG seedlings of *tip1-4* treated with either DMSO or 2-BP. **D**-**E**. Representative root hairs at the hair elongation zone of 4 DAG seedlings treated with either DMSO **(D)** or with 2-BP **(E)**. **F**-**G**. Root hair length **(F)** and width **(G)**. Results are means ± standard errors (SE), N = 4. Length or width of mature wild-type root hairs treated with DMSO was set as 1. Empty bars represent DMSO treatment while filled bars represent 2-BP treatment. Asterisks indicate significant difference (Student’s *t*-test, P < 0.05). Bars = 500 μm for **(A-C)**; 20 μm for **(D-E)**.

We examined the effect of inhibiting protein palmitoylation on the initiation and polar growth of root hairs. Application of 10 μM 2-BP for 12 hrs did not cause a substantial change in primary root length (Figure 2A). However, compared to roots treated with DMSO, those treated with 2-BP showed a much expanded region of root hair initiation (Figure 2A), suggesting inhibited root hair elongation. Indeed, the polar growth of root hairs was significantly affected by 2-BP treatment, such that root hairs were shorter and wider than those treated with DMSO at the maturation zone (Figure 2D,E,F,G). In addition, 2-BP caused extensive vacuolation in growing root hairs, compared to root hairs treated only with DMSO (Figure 2D,E).

**Figure 3:**
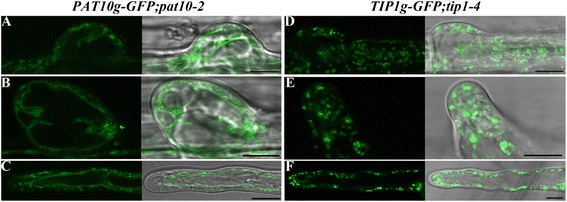
**TIP1 and PAT10 localize at different endomembrane compartments in root hairs. A**-**C**. Representative initiating root hair **(A)**, elongating root hair **(B)**, or mature root hair **(C)** of 4 DAG *PAT10g-GFP;pat10-2* transgenic seedlings. **D**-**F**. Representative initiating root hair **(D)**, elongating root hair **(E)**, or mature root hair **(F)** of 4 DAG *TIP1g-GFP;tip1-4* transgenic seedlings. Bars = 10 μm.

Most Arabidopsis *PAT*s represented on the ATH1 Chip [32] are expressed in root hairs or pollen tubes (Additional file 1: Figure S2). Indeed, *tip1* mutants exhibited defective growth in both root hairs and pollen tubes [18-20]. TIP1 was shown to be Golgi-localized [17] by using transient expression in tobacco epidermal cells. However, transient heterogeneous expression with strong constitutive promoters does not always reflect the native localization of proteins, as is the case for PAT10 [12,17]. To determine its native localization, we introduced a *TIP1* genomic fragment-GFP translational fusion driven by its native promoter (*TIP1*g-GFP) into *tip1-4*, a novel null mutant (Additional file 1: Figure S3). *TIP1*g-GFP fully restored the root hair defects of *tip1-4* (Figure 3D,E,F), indicating that the GFP fusion did not interfere with its functionality. To verify that the punctate vesicles labeled by TIP1 were of Golgi identity, we applied the lipophilic dye FM4-64 and the fungal toxin Brefeldin A (BFA) to *TIP1*g-GFP;*tip1-4* roots. FM4-64 enters cells via endocytic trafficking and sequentially labels *trans*-Golgi network/early endosomes (TGN/EE), prevacuolar compartment/multivesicular bodies (PVC/MVB), then finally reaches the tonoplast [33]. BFA interferes with the activity of Arf GTPases and its application resulted in the formation of so-called BFA compartments with a TGN/EE core surrounded by aggregates of Golgi [34]. FM4-64 uptake together with BFA treatment confirmed the localization of TIP1 at the Golgi (Additional file 1: Figure S4). To find out whether *TIP1* played a dominant role in root hair growth, we applied 2-BP to *tip1-4* roots and analyzed root hair morphology. There were slight but not significant morphological changes to root hair length and width in 2-BP-treated *tip1-4* (Figure 2C,D,E,F), suggesting that TIP1 is a major PAT functioning in root hairs.

*PAT10* is also expressed in root hairs (Additional file 1: Figure S2). However, it was unclear whether *PAT10* played a role in root hair growth [12]. In *PAT10*g-GFP;*pat10-2*, PAT10 was localized at the tonoplast of root hairs at all stages (Figure 3A,B,C). We therefore analyzed the root hair morphology of *pat10-2* in the absence or presence of 2-BP. Root hair initiation and polarity was not affected, as hair width was comparable between WT and *pat10-2* (Figure 2G). However, root hair length was significantly reduced by *PAT10* loss-of-function (Figure 2F). Treatment of 2-BP resulted in similar defects in *pat10-2* as in wild type (Figure 2A), i.e. root hair initiation was substantially inhibited (Figure 2B). These results suggest that protein palmitoylation is important for root hair growth, with TIP1 plays a major role and other PATs, such as PAT10, also participating.

### 2-BP disrupts actin MF polymerization and the asymmetric PM localization of PIP_2_

**Figure 4:**
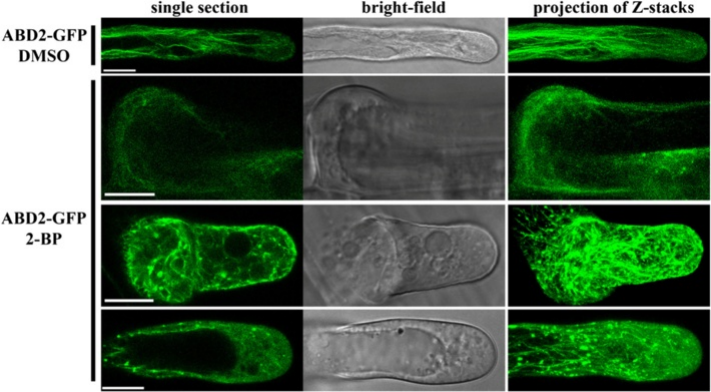
**2-BP induced fragmentation and cross-linking of actin MF in root hairs.** 4 DAG seedlings of *Pro*_*35S*_:GFP-ABD2-GFP transgenic seedlings were treated with DMSO or with 10 μM 2-BP for 2–3 hr before imaging. 18 to 20 root hairs at different stages were examined and representative images are shown. Single section indicates one optical section taken at the mid-plane of a root hair. For each root hair shown, twenty 1 μm optical sections were superimposed to generate the projection of Z-stacks. Bars = 10 μm.

Because 2-BP treatment significantly affected the polar growth of root hairs, we explored the underlying mechanisms by examining the effects of 2-BP on critical intracellular activities during root hair growth such as the dynamic polymerization of actin MF [21-24] and the asymmetric PM distribution of PIP_2_ [35-37]. To analyze actin MF dynamics, we treated Arabidopsis transgenic plants expressing GFP-ABD2-GFP, which specifically labels actin MF [24,38-40] with either 10 μM 2-BP or DMSO and examined the pattern of actin MF in root hairs. In root hairs treated with DMSO, longitudinal or slightly helical actin cables extended to the subapical region from the base while short actin bundles as indicated by punctate filamentous signals were present in the apical region where active growth occurred (Figure 4). By contrast, treatment with 2-BP caused fragmentation as well as extensive cross-linking of actin MF (Figure 4). As a result, few longitudinal actin cables were observed in 2-BP-treated bulging root hairs (Figure 4). Instead, numerous short actin filaments formed a mesh-like network extending to the apical region (Figure 4). In elongating root hairs under 2-BP treatment, actin cables along the root hair shank were dotted with punctate actin aggregates (Figure 4). These effects occurred as quickly as 2–3 hrs after 2-BP treatment, indicating the sensitivity of the dynamic polymerization of actin MF. Treatment of root hairs with the actin MF depolymerization drug Latrunculin B (LatB) indicated that depolymerization of actin MF did result in punctate aggregates (Additional file 1: Figure S5). However, LatB treatment did not cause extensive cross-linking of actin MF in root hairs (Additional file 1: Figure S5), in contrast to those treated with 2-BP (Figure 4). These results suggest that the effect of 2-BP on actin MF polymerization is complex.

**Figure 5:**
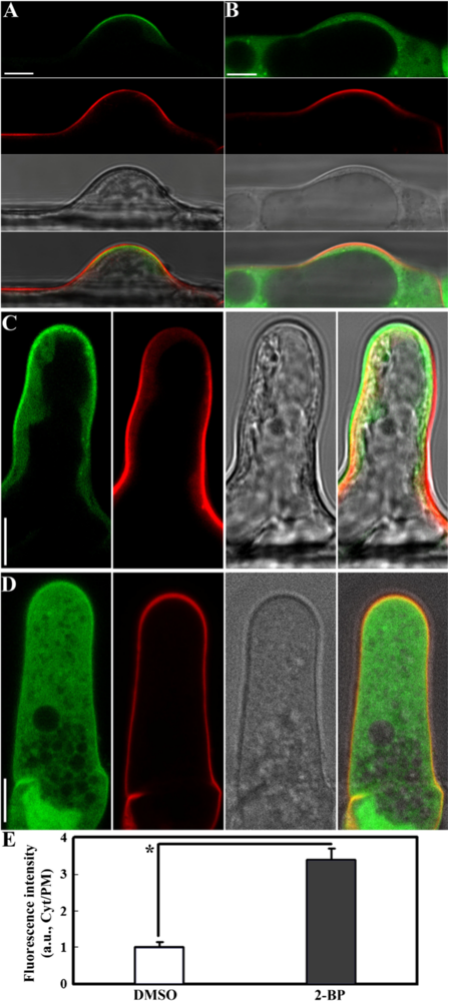
**2-BP treatment re-distributed the PIP**_**2**_ **sensor from the PM to the cytoplasm in root hairs. A**. DMSO-treated root hairs expressing the PIP_2_ sensor (green) at the initiating stage. **B**. 2-BP-treated root hairs expressing the PIP_2_ sensor at the initiating stage. **C**. DMSO-treated root hairs expressing the PIP_2_ sensor at the elongating stage. **D**. 2-BP-treated root hairs expressing the PIP_2_ sensor at the elongating stage. **E**. Ratio of fluorescence signals. a.u. stands for arbitrary fluorescence units. Cyt/PM indicates the ratio of cytoplasmic to the plasma membrane signal. Results are means ± standard deviation (SD, n = 30). Asterisk indicates significant difference (Student’s *t*-test, P < 0.01). Root hairs were stained with the fluorescence dye propidium iodide (red) to outline cell shape. Corresponding bright-field images are shown together with merges of different channels. Bars = 7.5 μm.

To determine the effect of 2-BP on the asymmetric PM localization of PIP_2_, we treated a P15Y fluorescence sensor line [41] with 2-BP or with DMSO. The P15Y sensor line expresses a *Pro*_*UBQ10*_-driven PIP_2_-binding TUBBY-C fused with CITRINE [41]. As shown by other PIP_2_ sensors [42], PIP_2_ was asymmetrically localized at the PM of initiation sites in trichoblasts (Figure 5A). During hair elongation, PIP_2_ maintained its asymmetric PM localization at the apical region (Figure 5B). Application of 2-BP significantly redistributed fluorescence signals from the PM to cytosol (Figure 5C,D,E), suggesting abolished PIP_2_ at the PM. For root hairs either at the initiation stage (Figure 5C) or at the elongating stage (Figure 5D), PIP_2_ was detected mostly in the cytoplasm and hardly at all at the PM. The residual signals at the PM were uniform (Figure 5D) rather than asymmetric (Figure 5B).

**Figure 6:**
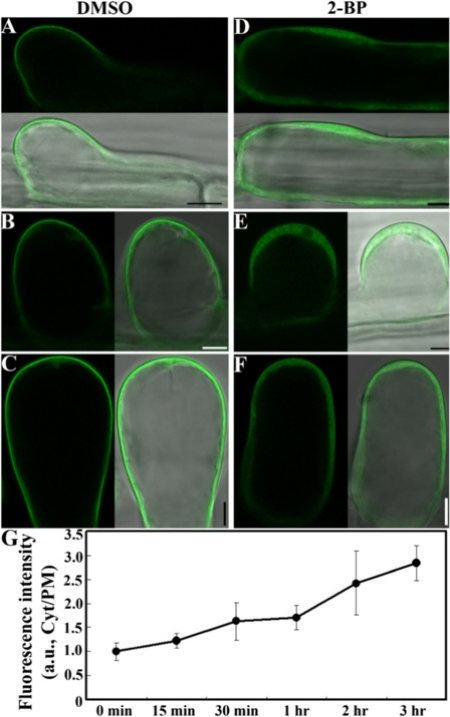
**2-BP treatment relocalizes ROP2 from the PM to the cytoplasm. A**-**C**. Representative initiating root hair **(A)**, elongating root hair **(B)**, or mature root hair **(C)** of 4 DAG *Pro*_*E7*_: GFP-ROP2 transgenic seedlings treated with DMSO for 3 hr. **D**-**F**. Representative initiating root hair **(D)**, elongating root hair **(E)**, or mature root hair **(F)** of 4 DAG *Pro*_*E7*_: GFP-ROP2 transgenic seedlings treated with 2-BP for 3 hr. **G**. Ratio of fluorescence signal intensity indicating the relative distribution of ROP2 in the cytoplasm and PM (Cyt/PM). a.u. stands for arbitrary fluorescence units. Results are means ± SD, n = 16. Bars = 7.5 μm.

The effect of 2-BP on the dynamic polymerization of actin MF and PIP_2_ distribution indicated polarity defects. Because actin MF and PIP_2_ distribution in tip-growing plant cells are regulated by or associated with ROP GTPases [43,44] that are subjected to palmitoylation [8-10], we wondered if 2-BP treatment could redistribute ROP GTPases into the cytoplasm or cause an uniform localization at the PM rather than the apex-restricted PM localization in root hairs [43,45]. To this end, we applied either DMSO or 2-BP to *Pro*_*E7*_:GFP-ROP2 transgenic seedlings in which ROP2, the key ROP GTPase regulating root hair growth [43], was driven by a root hair-specific promoter [46]. ROP2 was concentrated at the PM of hair initiation sites in trichoblasts of *Pro*_*E7*_:GFP-ROP2 transgenic seedlings treated with DMSO (Figure 6A), as previously reported [43,45,47]. Expression of *ROP2* caused root hair bulging (Figure 6A,B,C). Likely due to the overexpression effect, ectopic ROP2 signals were detected along the PM as well as in the cortical cytoplasm (Figure 6A,B,C). By contrast, 2-BP treatment induced rapid re-localization of ROP2 from the PM to the cytoplasm (Figure 6D,E,F). Significant differences were observed as early as 30 min after 2-BP treatment (Figure 6G).

### RabA4b-positive post-Golgi secretion was impaired by 2-BP in root hairs

**Figure 7:**
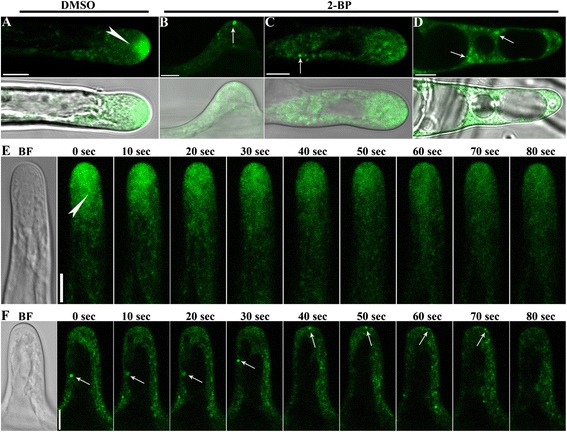
**2-BP treatment interfered with the dynamics of RabA4b-positive secretory vesicles. A**. Distribution of RabA4b-positive vesicles in a growing root hair of 4 DAG RFP-RabA4b transgenic seedlings treated with DMSO for 2 hr. The arrowhead points at the base of the clear zone where RabA4b-positive vesicles form an inverted cone. Below is merge of fluorescence and bright field images. **B**-**D**. Distribution of RabA4b-positive vesicles in a root hair right after initiation **(B)**, a growing root hair **(C)**, or an arrested root hair **(D)** of 4 DAG RFP-RabA4b transgenic seedlings treated with 10 μM 2-BP for 2 hr. The arrows point at enlarged vesicles positive for RabA4b. Below are the merges of fluorescence and bright field images. **E**-**F**. RFP-RabA4b fluorescence was visualized in root hairs treated with DMSO **(E)** or 10 μM 2-BP **(F)** for 2 hr using time-lapse confocal fluorescence microscopy. Left-most are the bright-field images. The arrowhead points to the base of the clear-zone. The arrows follow the moving track of a single large vesicle over time. Bars = 7.5 μm.

Polarized growth requires regulated exocytosis to deliver building materials for membranes and cell walls. In Arabidopsis root hairs, RabA4b-mediated secretory vesicles form an inverted cone-shaped pattern critical for polarized growth [26,27,29,30]. To determine the effect of 2-BP on RabA4b-positive post-Golgi secretion, we applied either 2-BP or DMSO to 4 DAG seedlings transformed with *Pro*_*35S*_:RFP-RabA4b. As reported previously [26,27,29,30], RFP-RabA4b was dynamically distributed to the apical cytoplasm in the form of an inverted cone with a trail in growing root hairs, which was not disturbed by DMSO (Figure 7A). Such a distribution pattern was dynamically maintained as long as root hairs grew (Figure 7E, Additional file 2: Movie S1). Application of 2-BP incurred two noticeable effects in root hairs: it disrupted the tip-focused inverted cone pattern and caused aggregation of RabA4b-positive vesicles (Figure 7B-D,F, Additional file 3: Movie S2). The effects of 2-BP were observed as early as 2 hr after treatment (Figure 7B-D), suggesting that post-Golgi secretory trafficking was sensitive to the inhibition of protein palmitoylation. Disruption of the tip-focused RabA4b-distribution pattern correlated with the growth kinetics of root hairs, in that RabA4b was more concentrated in the apical region than in the shank region in growing root hairs (Figure 7C) whereas completely dissipated into punctates in non-growing root hairs (Figure 7D). Despite the disruption on RabA4b-positive secretory trafficking, by following single aggregates during time-lapse confocal fluorescence microscopy, we observed that at least some vesicles were able to be exocytosed (Figure 7F).

### 2-BP inhibits endocytic and vacuolar trafficking

**Figure 8:**
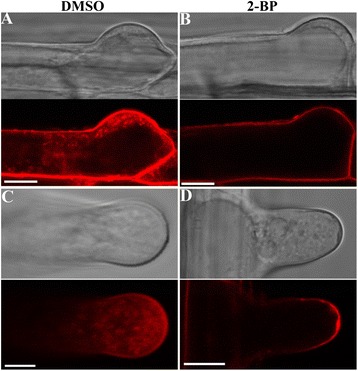
**2-BP inhibited endocytosis in root hairs. A**. FM4-64 uptake in initiating root hairs pre-treated with DMSO for 2 hr. **B**. FM4-64 uptake in initiating root hairs pre-treated with 10 μM 2-BP for 2 hr. **C**. FM4-64 uptake in elongating root hairs pre-treated with DMSO for 2 hr. **D**. FM4-64 uptake in elongating root hairs pre-treated with 10 μM 2-BP for 2 hr. Images shown are representative of 18–25 root hairs analyzed in three independent experiments. Bars = 10 μm.

The disrupted RabA4b distribution pattern by 2-BP prompted us to test whether endocytosis was affected because polarized growth requires balanced exocytosis and endocytosis to maintain the dynamic integrity of the cell membranes and walls. FM4-64 enters into plant cells through the PM by endocytosis and eventually reaches the tonoplast [33]. To determine whether 2-BP interfered with endocytic trafficking, we pre-treated 4 DAG seedlings with 10 μM 2-BP or DMSO for 2 hrs before pulse-labeling the roots with 4 μM FM4-64. In both initiating (Figure 8A) and elongating root hairs (Figure 8C) pre-treated with DMSO, FM4-64 was internalized from the PM into the TGN/EE (Additional file 4: Movie S3). By contrast, no cytosolic vesicles were observed in root hairs pre-treated with 2-BP during the time course of the experiment (Additional file 5: Movie S4) either in initiating root hairs (Figure 8B) or in elongated root hairs (Figure 8D), indicating complete inhibition of endocytosis.

**Figure 9:**
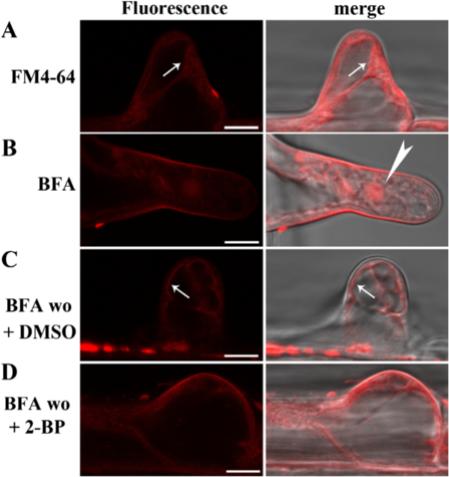
**2-BP interfered with vacuolar trafficking. A**-**D**. 4 DAG WT seedlings were pulse-labeled with FM4-64, washed and incubated for 30 min **(A)**, or pulse-labeled with FM4-64 followed by 30 min incubation with 1/2 MS medium supplemented with 50 μM BFA **(B)**. BFA-treated seedlings were then washed with 1/2 MS medium supplemented with either DMSO (BFA wo + DMSO) **(C)** or 2-BP (BFA wo + 2-BP) **(D)**. Arrows point at the tonoplat labeled by FM4-64. Arrowhead indicates the BFA compartment. Results are representative of 20 root hairs for each treatment. Bars = 7.5 μm.

Endocytic trafficking starts at the PM and ends at the vacuole. To find out whether vacuolar trafficking was influenced by 2-BP treatment, we followed the endocytic trafficking of FM4-64 to the tonoplast. FM4-64 labeled both cytosolic vesicles and the tonoplast after 30–40 min uptake in root hairs (Figure 9A). Because BFA treatment caused aggregation of FM4-64-labeled TGN/EE into BFA compartments (Figure 9B), we reasoned that a BFA washout would allow us to examine the process of vacuolar trafficking from TGN/EE via PVC/MVB to vacuoles. In root hairs treated with DMSO, BFA washout led to the labeling of FM4-64 of the tonoplast (Figure 9C), indicating undisturbed trafficking from the TGN/EE to vacuoles. However, in the presence of 2-BP, BFA washout resulted in dissipation of FM4-64 signals from the BFA compartments (Figure 9D). Furthermore, FM4-64 was redistributed mostly to the PM rather than to the tonoplast (Figure 9D), suggesting that 2-BP caused mis-sorting of vesicles originally destined to vacuoles.

To further support the idea that 2-BP inhibited vacuolar trafficking, we applied either 2-BP or DMSO to root hairs expressing YFP-2XFYVE, which binds specifically to PI3P [41]. Because PI3P goes to vacuoles for degradation through vacuolar trafficking routes from TGN/EE to PVC/MVB, we reasoned that this would serve as a good biosensor for vacuolar trafficking [48]. Indeed, 2-BP but not DMSO induced the formation of ring-shaped compartments positive for PI3P, to an extent similar to but less substantial than that caused by wortmannin (Additional file 1: Figure S6), through which PVC/MVBs on their way to vacuoles fuse to form ring-shaped compartments [49]. These results indicated that vacuole trafficking through the TGN/EE and PVC/MVBs was compromised by 2-BP.

## Discussion

As a reversible post-translational modification, protein palmitoylation has been extensively studied in polarized cell growth in metazoans [6]. By using pharmacological and genetic approaches, we demonstrate that protein palmitoylation, contributed primarily by the Golgi-localized TIP1 (Figure 3, Additional file 1: Figure S4) and secondarily by PATs from other endomembrane compartments such as vacuoles (Figure 3), plays a key role in the polar growth of root hairs. By using the tonoplast-cytoplasm partition of CBL2 as an indicator for effective inhibition of palmitoylation, we determined the application regime of 2-BP on root hair growth (Figure 1). 2-BP has been used extensively in yeast and metazoans [1,31] but rarely in plants [11,12,18]. Treatment with 2-BP resulted in shorter and wider root hairs (Figure 2), suggesting compromised hair elongation and polarity due to inhibited palmitoylation. Functional loss of *PAT10* resulted in shorter root hairs but affected width (Figure 2) indicate that *PAT10* functions in hair elongation but not in polarity control. Treatment of 2-BP resulted in an additional reduction in hair length in *pat10-2* (Figure 2), suggesting that other *PAT*s are involved in hair elongation. In contrast to *pat10-2*, in *tip1-4* neither hair length or width was significantly affected by 2-BP (Figure 2), suggesting that TIP1 is the primary PATs controlling root hair elongation and polarity. However, 2-BP does induce an expansion of root hair initiation zone in *tip1-4* as in wild type (Figure 2), indicating that other PATs also contribute to hair elongation, at least in specific context. Indeed, multiple *PAT*s are expressed in root hairs (Additional file 1: Figure S2) besides *TIP1* and *PAT10* and their diverse subcellular distributions as revealed by a transient expression assay [17] hinted at a complex effect of protein palmitoylation on root hair growth.

Root hair growth requires dynamic distribution of polarity proteins, among which ROP GTPases [43,45-47] are crucial. As their yeast and metazoan counterparts [6,7], ROP GTPases are palmitoylated proteins whose membrane distribution and activities rely on their palmitoylation status [9,10]. We showed that 2-BP causes a significant translocation of ROP2 from the PM to the cytoplasm (Figure 6), indicating membrane dissociation due to reduced palmitoylation. However, palmitoylation of several ROP GTPases was shown to be crucial for their partitioning among membrane microdomains rather than between the PM and the cytoplasm [9,10]. The discrepancy could be due to the specific property of tip-growing root hair cells, in which heterogeneity of the PM is spatially reflected on a much larger scale than in microdomains of non-polar growing cells [50].

As central regulators of polarized cell growth in plants [50,51], ROP GTPases play critical roles in multiple intracellular activities, most importantly, the dynamic polymerization of actin MF [43] that is crucial for maintaining polar growth in root hairs [21-24]. Treatment of 2-BP cause substantial fragmentation of actin MF (Figure 4), indicating impaired actin MF polymerization. However, the effect of 2-BP is different from that induced by the actin MF depolymerization drug LatB (Additional file 1: Figure S4) such that 2-BP results in extensive cross-linking as indicated by strong puncta at the interaction of several short actin bundles (Figure 4). The dissociation of ROP GTPases from the PM of root hairs (Figure 6) only partially explains the effect because interfering with ROP activities in root hairs by expressing a dominant negative ROP2 [43] does not result in the dis-organized actin MF network. A more likely scenario is that other palmitoylated proteins than ROPs may regulate actin MF dynamics in root hairs, as was reported for some receptor kinases during neuronal growth [6].

Polar growth of root hairs requires restricted delivery of secretory vesicles [28,52]. RabA4s are critical for post-Golgi secretion in root hairs [26,27,29,30] by forming an inverted cone-shaped vesicle stream to deliver materials for growth. We showed that 2-BP dissipates the tip-focused distribution pattern of RabA4b-positive post-Golgi secretory vesicles and caused their aggregation (Figure 7). Because post-Golgi vesicles rely on dynamic polymerization of actin MF for their motility and possibly for their directionality in root hairs [24,28], the disrupted actin MF network due to 2-BP (Figure 4) may have indirectly resulted in the impaired secretion (Figure 7).

Endocytosis not only retrieves excess materials delivered from exocytosis to maintain cellular homeostasis but also mediates the membrane distribution of key signaling proteins during polar growth [50]. As a key signaling molecule, PIP_2_ was recently shown to regulate clathrin-mediated endocytosis [53]. By using a fluorescence probe specific for PIP_2_ [41], we showed that 2-BP causes a rapid loss of PIP_2_ at the PM (Figure 5), which correlates with the complete inhibition of endocytosis by 2-BP pre-treatment (Figure 8). In addition to internalization from the PM, vacuolar trafficking from the TGN/EE is also compromised by 2-BP (Figure 9, Additional file 1: Figure S6). Rather than proceeding to the tonoplast from the TGN/EE, FM4-64 instead traffics to the PM (Figure 9), indicating defective vacuolar trafficking. By using a fluorescence probe specific for PI3P, we showed that 2-BP caused homotypic fusion of PVC/MVBs rather than fusion of PVC/MVBs to vacuoles (Additional file 1: Figure S6). The dramatic responses of membrane trafficking to 2-BP suggests that key proteins regulating membrane trafficking in plant cells are controlled by protein palmitoylation. SNAREs are critical components in vesicle trafficking machinery critical for selective vesicle fusion [48]. Many SNAREs are modified by palmitoylation in yeast and metazoans and such palmitoylation might be evolutionarily conserved [2,4]. The Arabidopsis genome encodes a large number of SNAREs [48] that are localized differentially at Golgi and post-Golgi compartments [54]. Combining genetic analyses and dynamic subcellular targeting of these SNAREs may reveal important substrates of protein palmitoylation during root hair growth.

## Conclusions

As a reversible post-translational modification that often regulates subcellular targeting and activities of signaling proteins, protein palmitoylation has been demonstrated to be critical for polar growth in metazoans. By using genetic as well as pharmacological approaches, we show here that protein palmitoylation, regulated by protein S-acyl transferases from different endomembrane compartments such as Golgi and vacuole, is critical for the polar growth of root hairs in Arabidopsis. Inhibition of protein palmitoylation by application of 2-BP disturbed key intracellular activities in root hairs, including actin MF polymerization, the asymmetric distribution of PIP_2_, post-Golgi secretion, as well as endocytic trafficking. Although some of the effects were likely indirect, the cytological data reported here will contribute to a deep understanding of protein palmitoylation during tip growth in plants.

## Methods

### Plant materials and growth conditions

The T-DNA insertion line, SALK_089971C (*tip1-4*), was obtained from the Arabidopsis Biological Resource Center (ABRC, http://www.arabidopsis.org). Primers F1/R1 were used to characterize *TIP1* expression in *tip1-4. Arabidopsis thaliana* Col-0 ecotype was used as wild type. Arabidopsis plants were grown as described [12]. For seedlings growing on plates, surface-sterilized Arabidopsis seeds were grown on Murashige and Skoog basal medium with vitamins (MS) (Phytotechlab, http://www.phytotechlab.com/) except where noted. Plates were kept at 4°C in darkness for 4 days before being transferred to a growth chamber with a 16-h light:8-h dark cycle at 21°C. Transgenic plants were selected on MS medium supplemented with 30 μg/ml Basta salt (Sigma, http://www.sigmaaldrich.com/).

### Plasmid construction

All vectors were generated using the Gateway™ technology (Invitrogen). Entry vectors for the coding sequence of *CBL2* and the whole genomic sequence of *TIP1* including its native promoter were generated in the pENTRY/SD/D-TOPO (Invitrogen) using the primer pair ZP595/ZP596 for *CBL2* and ZP533/ZP534 for *TIP1*g. The destination vector *Pro*_*UBQ10*_:GW-RFP was generated by replacing the *Pro*_*35S*_ promoter with *Pro*_*UBQ10*_ using the primer pair ZP510/ZP511 with the *Spe*I/*Hind*III double digestion sites from a previously described destination vector [55]. *TIP1g*-GFP was generated by an LR reaction using a GW:GFP translation fusion destination vector [12] and the *TIP*1g entry vector. *Pro*_*E7*_:GFP-ROP2 was generated by a LR reaction using the *Pro*_*E7*_:GFP-GW destination vector and the entry vector for *ROP2* [46]. All PCR amplifications used Phusion^TM^ hot start high-fidelity DNA polymerase with the annealing temperature and extension times recommended by the manufacturer (Finnzyme). All entry vectors were sequenced and verified. The Bioneer PCR purification kit and the Bioneer Spin miniprep kit were used for PCR product recovery and plasmid DNA extraction, respectively. Primers are listed in (Additional file 1: Table S1).

### Quantification of root hair length and width

In the presence of 2-BP or DMSO, the region of root growth and root hair expansion was 1–1.5 mm distal from the primary root tip of 4 DAG seedlings and was thus chosen for length and width measurements. Images of that region were taken from individual seedlings using an Axio Observer A1 equipped with a CCD camera (Zeiss). Quantification of root hair length and width was performed according to previous descriptions [46] using ImageJ (http://rsbweb.nih.gov/ij/).

### Pharmacological treatments

Stock solutions of various inhibitors (Sigma) were prepared using DMSO as the solvent at the following concentrations: 50 mM 2-BP, 35 mM BFA, 20 mM CHX and 4 mM FM4-64. Stock solutions were diluted and added to 1/2 MS at designated final concentrations, i.e. 10–50 μM 2-BP, 50 μM BFA, 50 μM CHX, and 4 μM FM4-64. DMSO was similarly diluted for the controls. All experiments were repeated at least three times. Images and movies shown are representative of approximately 18–30 root hairs.

### Fluorescence labeling, quantification, and microscopy

Fluorescent images were captured using a Leica TCS SP5II confocal laser scanning microscope (Leica, Wetzlar, Germany) with a Plan-Neofluar 40×/1.3NA oil DIC objective or 63×/1.45NA oil DIC objectives. GFP-RFP double-labeled plant materials were captured alternately using line switching with the multi-track function (488 nm for GFP and 545 nm for RFP). Fluorescence was detected using a 505- to 550-nm band-pass filter for GFP and a 575- to 650-nm band-pass filter for RFP. YFP-RFP double-labeled plant materials were captured alternately using line switching with the multi-track function (514 nm for YFP and 545 nm for RFP). Fluorescence was detected using a 530- to 580-nm band-pass filter for YFP and a 575- to 650-nm band-pass filter for RFP. Post-acquisition image processing was performed with the LAS AF Lite image processing software (Leica). For quantification of PIP_2-(Cyt/PM)_, ROP2_-(Cyt/PM)_, and CBL2_-(Tonoplast/Cyt)_, regions of interest (ROIs) of same sizes were designated at the PM, the cytoplasm or the tonoplast. Average signal intensity at ROIs was measured using ImageJ and ratio of the cytoplasm to the PM (for PIP_2_), the PM to the cytoplasm (for ROP2) or the tonoplast to the cytoplasm (for CBL2) was calculated. All vacuolated areas were excluded from the cytoplasm ROI.

### Accession numbers

Arabidopsis Genome Initiative locus identifiers for the genes mentioned in this article are: At2g40990 for *PAT2*; At5g05070 for *PAT3*; At3g56930 for *PAT4*; At3g48760 for *PAT5*; At5g41060 for *PAT6*; At3g26935 for *PAT7*; At4g24630 for *PAT8*; At5g50020 for *PAT9*; At3g51390 for *PAT10*; At4g22750 for *PAT13*; At3g60800 for *PAT14*; At5g04270 for *PAT15*; At3g09320 for *PAT16*; At3g04970 for *PAT17*; At4g01730 for *PAT18*; At3g22180 for *PAT20*; At2g33640 for *PAT21*; At1g69420 for *PAT22*; At5g20350 for *TIP1/PAT24*; At5g55990 for *CBL2*; At1g12560 for *EXP7*; At1g20090 for *ROP2*; and At4g39990 for *RabA4b*.

## Additional files

Additional file 1:
**Six supplemental figures and their corresponding legends.**
Additional file 2:
**A movie showing dynamic distribution of RFP-RabA4b in a root hair treated with DMSO for 2 hr.**
Additional file 3:
**A movie showing dynamic distribution of RFP-RabA4b in a root hair treated with 10 μM 2-BP for 2 hr.**
Additional file 4:
**A movie showing uptake of FM4-64 in root hairs pre-treated with DMSO for 2 hr.**
Additional file 5:
**A movie showing uptake of FM4-64 in root hairs pre-treated with 10 μM 2-BP for 2 hr.**

## Abbreviations

2-BP: 2-bromopalmitate
BFA: Brefeldin A
CBL: Calcineurin B like proteins
DAG: Days after germination
DMSO: Dimethyl sulfoxide
ER: Endoplasmic reticulum
LatB: Latrunculin B
MF: Microfilaments
PAT: Protein S-acyl transferases
PIP_2_: Phosphatidylinositol (4,5)-bisphosphate
PM: Plasma membrane
PVC/MVB: Prevacuolar compartment/multivesicular bodies
SNAREs: *N*-ethylmaleimide-sensitive factor-activating protein receptors
TIP1: TIP GROWTH DEFECTIVE1
TGN/EE: *trans*-Golgi network/early endosomes
TM: Transmembrane
