# Supplementary material for: Protein palmitoylation is critical for the polar growth of root hairs in Arabidopsis
Source: BMC Plant Biol. 2015 Feb 13;15:50. doi: 10.1186/s12870-015-0441-5 (PMC4340681; doi:10.1186/s12870-015-0441-5)
Supplement: Additional file 1: — Six supplemental figures and their corresponding legends. [file 12870_2015_441_MOESM1_ESM.pdf]

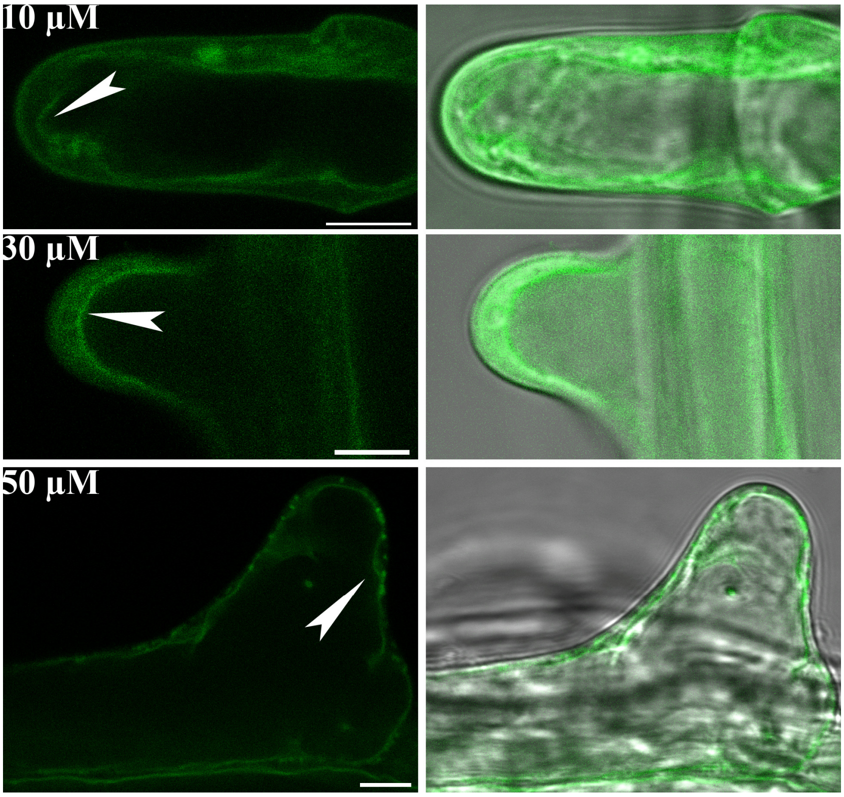

Figure S1. High concentrations of 2-BP induced vacuolation in root hairs without substantially redistributing CBL2 to the cytoplasm. 4 DAG seedlings of *Pro*$_{UBQ10}$:CBL2-RFP transgenic plants were treated with 10 µM, 30 µM or 50 µM 2-BP for 4 hr before visualization. On the right are merges of fluorescence and bright field images. Arrowheads point at the tonoplast. For each treatment, 18 root hairs were visualized and representative images are shown. Bars = 7.5 µm.

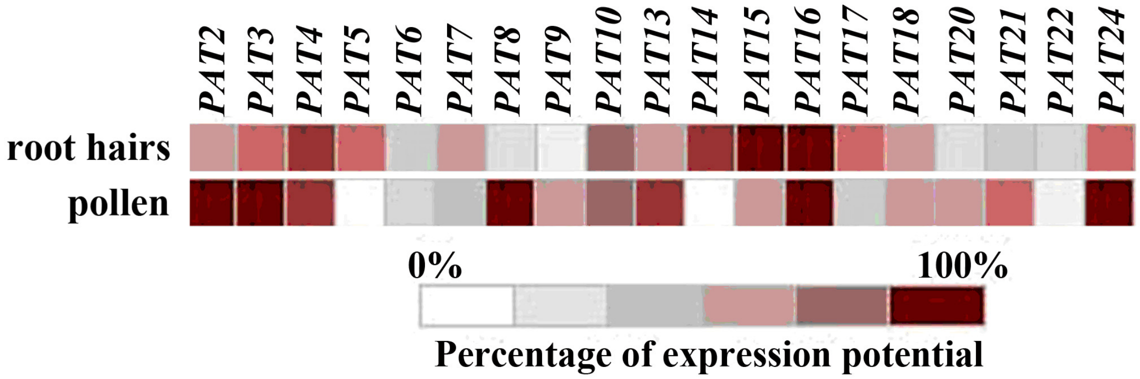

Figure S2. Expression of most Arabidopsis *PAT*s is detected in root hairs or pollen tubes. Data were obtained from the GENEVESTIGATOR database [1].

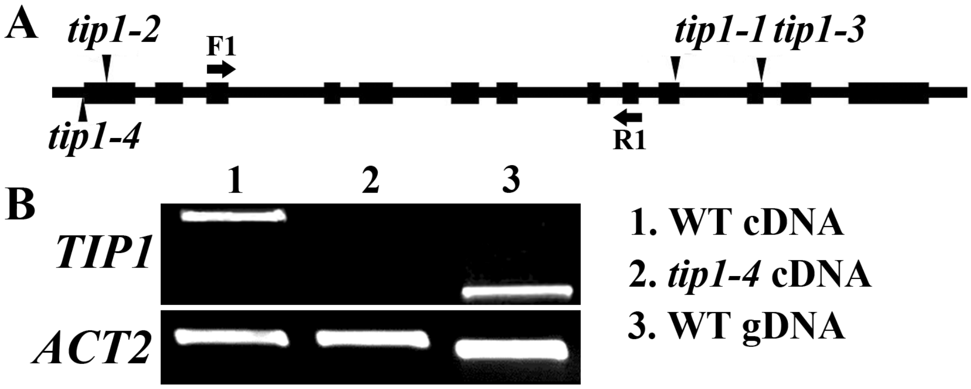

Figure S3. The *tip1-4* is a novel null mutant for *TIP1*. A. Schematic illustration of the *TIP1* genomic region and the T-DNA insertion site for *tip1-4*. **B.** Transcript analysis to verify that *tip1-4* is a null allele for *TIP1*. *ACTIN2* (*ACT2*) was used as the internal control.

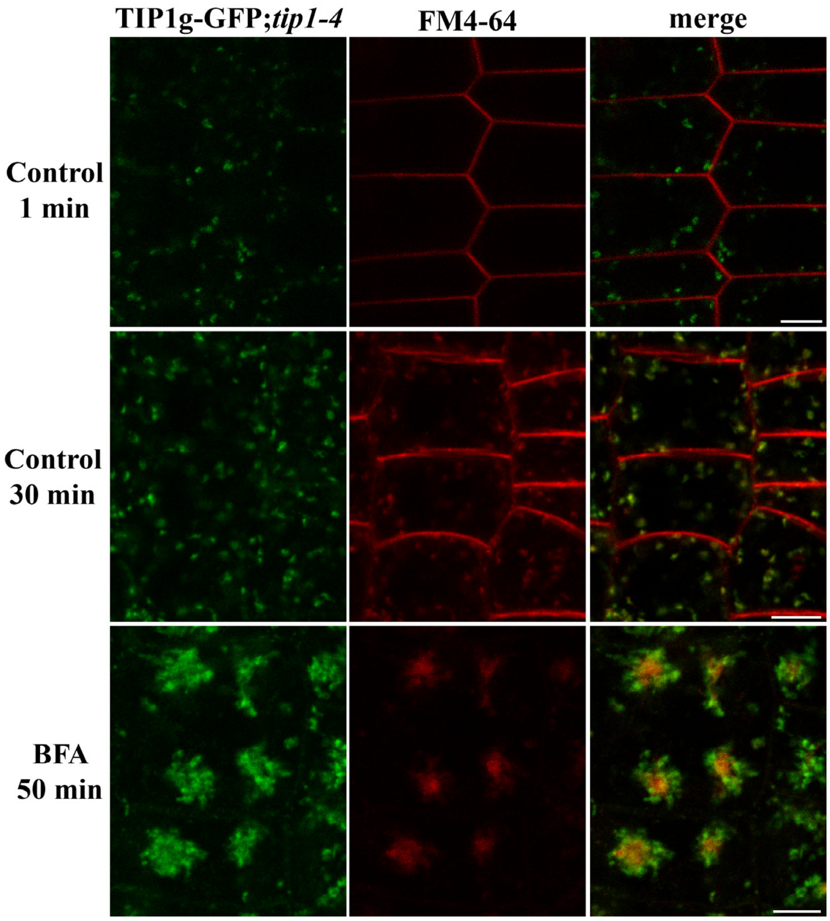

Figure S4. TIP1 localizes at the Golgi apparatus. 4 DAG seedlings of TIP1g:GFP in the *tip1-4* background were pulse-labeled with 4 µM FM4-64. Images were taken immediately after FM4-64 treatment (1 min), 30 min after FM4-64 labeling and washout (30 min), or 50 min BFA treatment after FM4-64 labeling and washout (BFA 50 min). Bars = 5 µm.

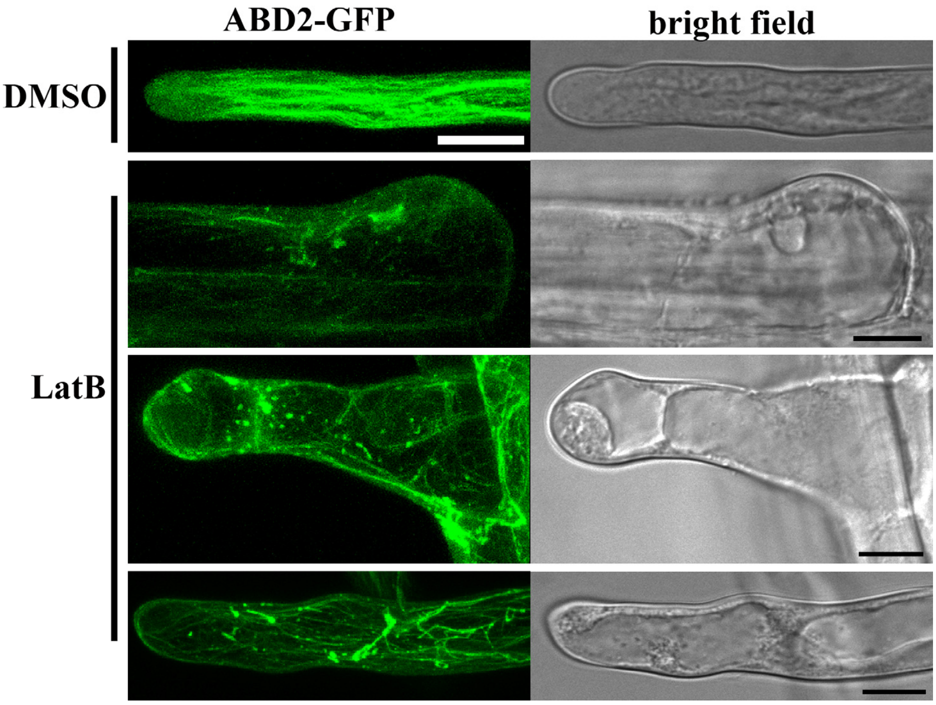

Figure S5. **Actin MF depolymerization in root hairs by LatB treatment.** Root hairs of 4 DAG *Pro₃₅ₛ*:GFP-ABD2-GFP transgenic seedlings were treated with DMSO or with 100 nM LatB for 3 hr before visualization. In total, 18 to 20 root hairs at different stages were examined and representative images are shown. Fluorescence images are superimposed from 20 of 1 μm optical sections. Bars = 10 μm.

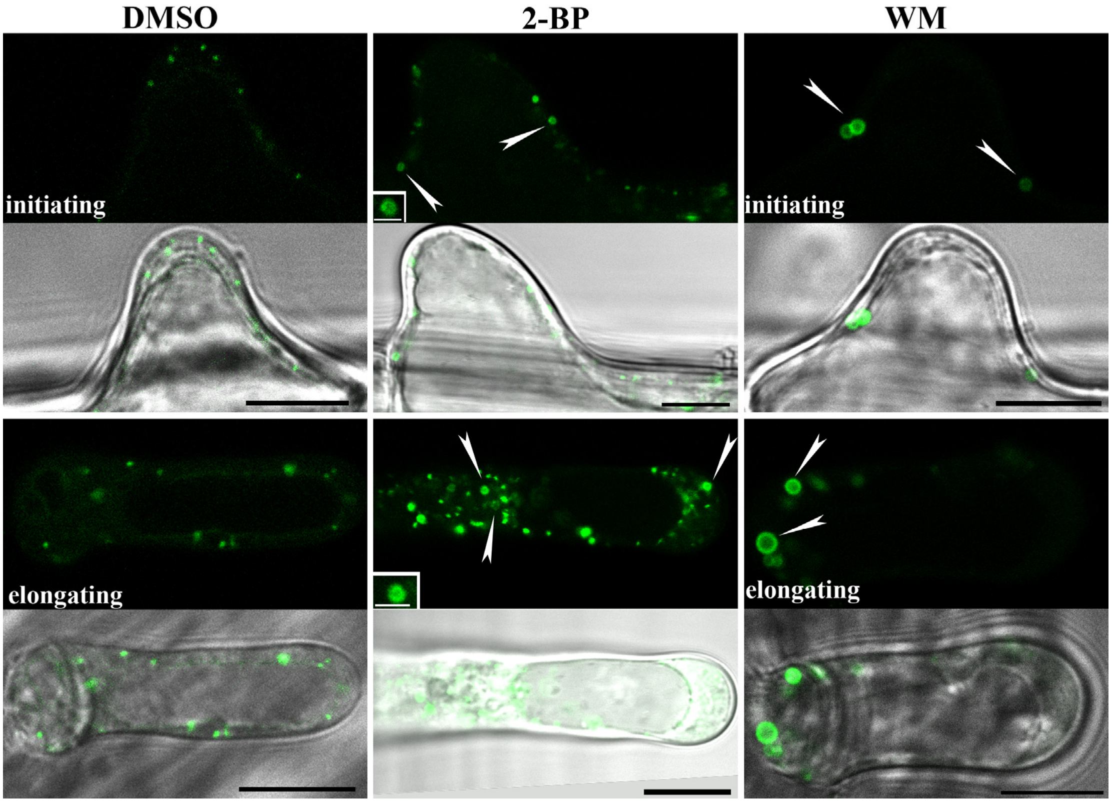

Figure S6. 2-BP induces fusion of PI3P-positive endosomes. 4 DAG seedlings expressing a YFP-fused PI3P probe were treated with DMSO, 33 μM wortmannin (WM) or 10 μM for 4 hr before visualization. 2-BP induced fusion of PI3P-positive endosomes both in initiating and elongating root hairs, similar to but to a less extent than WM. Arrowheads point at the ring-shaped compartments. Bars = 10 μm; 2 μm for insets.

Movie S1: Dynamic distribution of RFP-RabA4b in a root hair treated with DMSO for 2 hr. Images were taken using time-lapse confocal fluorescence microscopy at 2.5 sec interval. Video clip is playing at 3 frames/sec.

Movie S2: Dynamic distribution of RFP-RabA4b in a root hair treated with 10 μM 2-BP for 2 hr. Images were taken using time-lapse confocal fluorescence microscopy at 2.5 sec interval. Video clip is playing at 3 frames/sec.

Movie S3: Uptake of FM4-64 in root hairs pre-treated with DMSO for 2 hr. Images were taken using time-lapse confocal fluorescence microscopy at 2.5 sec interval. Video clip is playing at 3 frames/sec.

Movie S4: Uptake of FM4-64 in root hairs pre-treated with 10 μM 2-BP for 2 hr. Images were taken using time-lapse confocal fluorescence microscopy at 2.5 sec interval. Video clip is playing at 3 frames/sec.

Table S1. Oligos used in this study.

No. Oligo	5’ – 3’ sequence
ZP510	CACCATAAGCTTGTCGACGAGTCAGTAATAAACG
ZP511	ATACTAGTGACGTCTGTTAATCAGAAAAACTCAG
ZP533	CACCATATGTGTGAAAGTTGCTTAGACTTTCCC
ZP534	TGGAGATACAACAGGTCGGGT
ZP595	CACCATGTCGCAGTGCGTTGAC
ZP596	GGTATCTTCAACCTGAGAATGG
F1	ACTGCTTTTCTTTGTCACGTTGTC
R1	CAAGCAAAGCAGGATTGTTTAGCT

Zimmermann P, Hirsch-Hoffmann M, Hennig L and Gruissem W (2004) GENEVESTIGATOR. Arabidopsis microarray database and analysis toolbox. Plant Physiol 136: 2621-2632.
